# Transcriptional response of BALB/c mouse thyroids following *in vivo* astatine-211 exposure reveals distinct gene expression profiles

**DOI:** 10.1186/2191-219X-2-32

**Published:** 2012-06-14

**Authors:** Nils Rudqvist, Toshima Z Parris, Emil Schüler, Khalil Helou, Eva Forssell-Aronsson

**Affiliations:** 1 Department of Radiation Physics, Institute of Clinical Sciences, Sahlgrenska Cancer Center, Sahlgrenska Academy, University of Gothenburg, Sahlgrenska University Hospital, Gothenburg, SE-413 45, Sweden; 2 Department of Oncology, Institute of Clinical Sciences, Sahlgrenska Cancer Center, Sahlgrenska Academy, University of Gothenburg, Sahlgrenska University Hospital, Gothenburg, SE-413 45, Sweden

**Keywords:** Astatine-211, Radiobiology, Normal tissue damage, Gene expression, Radionuclide therapy

## Abstract

**Background:**

Astatine-211 (^211^At) is an alpha particle emitting halogen with almost optimal linear energy transfer for creating DNA double-strand breaks and is thus proposed for radionuclide therapy when bound to tumor-seeking agents. Unbound ^211^At accumulates in the thyroid gland, and the concept of basal radiation-induced biological effects in the thyroid tissue is, to a high degree, unknown and is most valuable.

**Methods:**

Female BALB/c nude mice were intravenously injected with 0.064 to 42 kBq of ^211^At, resulting in absorbed doses of 0.05 to 32 Gy in the thyroid gland. Thyroids were removed 24 h after injection; total RNA was extracted from pooled thyroids and processed in triplicate using Illumina MouseRef-8 Whole-Genome Expression Beadchips.

**Results:**

Thyroids exposed to ^211^At revealed distinctive gene expression profiles compared to non-irradiated controls. A larger number of genes were affected at low absorbed doses (0.05 and 0.5 Gy) compared to intermediate (1.4 Gy) and higher absorbed doses (11 and 32 Gy). The proportion of dose-specific genes increased with decreased absorbed dose. Additionally, 1.4 Gy often exerted opposite regulation on gene expression compared to the other absorbed doses. Using Gene Ontology data, an immunological effect was detected at 0.05 and 11 Gy. Effects on cellular response to external stress and cell cycle regulation and proliferation were detected at 1.4 and 11 Gy.

**Conclusions:**

Conclusively, the cellular response to ionizing radiation is complex and differs with absorbed dose. The response acquired at high absorbed doses cannot be extrapolated down to low absorbed doses or vice versa. We also demonstrated that the thyroid - already at absorbed doses similar to those obtained in radionuclide therapy - responds with expression of a high number of genes. Due to the increased heterogeneous irradiation at low absorbed doses, we suggest that this response partly originates from non-irradiated cells in the tissue, i.e., bystander cells.

## Background

Since the first measurement of clonogenic cell survival of HeLa cells exposed to X-rays in 1955 [1] and up to the early 1990s, radiobiological research has mainly focused on cell death and survival with an approach from the target-cell hypothesis. It was believed that the direct killing of parenchymal and vascular endothelial cells successively led to organ failure [2,3]. In addition, the time interval between exposure and manifestation of late normal tissue effects was considered to be ‘silent’, without any signs of tissue damage. For acute normal tissue damage, the target-cell hypothesis is still viable; however, it is not as reliable for modeling late normal tissue damage [4]. The discovery of early radiation-induced cytokine cascades in 1995 was a major step towards new concepts in radiobiology [5]. These findings, together with the identification of the bystander effect and studies of late side effects such as radiation-induced fibrosis, sequentially lead to a paradigm shift where the basal mechanisms of the cellular and molecular response to ionizing radiation now receive much focus [2,5,6]. Now, the cellular response to ionizing radiation may be seen as an orchestrated reaction where cell loss and gene expression both play important roles.

Astatine-211 (^211^At), an α-decaying radiohalogen, is considered to be an optimal therapeutic radionuclide with a mean dose-average linear energy transfer of 98.8 keV/μm, [7]. ^211^At-labeled tumor-seeking agents have been used in trials on both animals and humans [8-11]. However, metabolism of ^211^At-labeled substances involves release of ^211^At and uptake in, e.g., the thyroid gland. It has been shown that, while iodine is primarily transported into the thyroid follicle cells through the sodium-iodide symporter (NIS), astatine transport is less dependent on NIS [12].

The function of a cell is regulated by the expression of its genes. The level of gene expression is regulated by signal transduction pathways, which are biochemical signals that may originate within and/or outside the cell. A snapshot of cellular activity in response to external stimuli (e.g., ionizing radiation) can be obtained by measuring gene expression at the transcriptome level. In the present study, this is performed using high-throughput microarray analysis.

Earlier studies of radiation-induced transcriptional changes have mainly been performed *in vitro* in a controlled environment [13,14]. Few *in vivo* studies have been reported from animal experiments. Significant differences in gene expression signatures were observed for X-ray-exposed mouse brain, rectum, and kidney, varying between the different tissue types and time after exposure [15].

The aim of this study was to investigate *in vivo* thyroidal response in mice to ^211^At exposure by using gene expression analysis to determine potential dose–response relationships. Also, we wanted to validate genes suggested as biomarkers for radiation damage.

## Methods

### Radionuclides and radioactive measurements

^211^At was produced at the Cyclotron and PET Unit at Rigshospitalet in Copenhagen, Denmark, using the ^209^Bi(α,2n)^211^At reaction, and free ^211^At was prepared as described previously [16]. A gamma counter (Wallac 1480 Wizard® 3", Wallac Oy, Turku, Finland) was used to measure the activity in stock solutions and syringes before and after injection.

### Absorbed-dose calculations

The mean absorbed dose was calculated according to conventional Medical Internal Radiation Dose formalism:

(1) $${\bar{D}}_{\text{thyroid}}=\frac{{Ã}_{\text{thyroid}}\;\text{×}\;\sum n_{\text{i}}E_{\text{i}}\;\text{×}\;{\text{ϕ}}_{\text{i}}}{m_{\text{thyroid}}}$$

where *Ã*_thyroid_ is the cumulated activity in the thyroid gland, *n*_i_ is the yield of the radiation *i* with energy *E*_i_, ϕ_i_ is the absorbed fraction for radiation *i* in the target tissue, and *m*_thyroid_ is the mass of the thyroid [17].

The cumulated activity was calculated using previously published biodistribution data, where percentage of injected activity were given at *t* = 0, 1, 2, 4, 6, and 24 h after injection for a number of organs [18]. Then, to derive the cumulated activity, the trapezoidal rule was used to interpolate between two consecutive time points. Consequently, the relationship between cumulated activity and injected activity (IA) was determined to be *Ã* = 2,100*IA. The absorbed fraction was set to 1 as the mean range for alpha particles emitted from ^211^At is about 65 μm, and a standard thyroid mass of 3 mg was used (equal to roughly 3 μl) [19]. Furthermore, the absorbed-dose calculations only included energy deposited from alpha particles emitted from ^211^At and its daughter nuclide ^211^Po located in the thyroid.

### Animal experiment

Twenty-one female BALB/c nude mice aged 6 months were divided into six groups and weighed. The mice in five groups were intravenously injected in the tail vein with 0.064, 0.64, 1.8, 14, and 42 kBq ^211^At diluted in phosphate-buffered saline (pH 7). The sixth group was used as controls and treated with an empty syringe. The animals were killed after 24 h by cardiac puncture under anesthesia with pentobarbitalnatrium. The thyroids were collected, immediately snap-frozen in liquid nitrogen, and stored at −80°C until analysis. The absorbed doses delivered to the thyroids were 0.05, 0.5, 1.4, 11, and 32 Gy during 24 h.

### Gene expression analysis

Thyroid tissue from animals in the same group was pooled, and total RNA was extracted using the RNeasy Lipid Tissue Mini Kit (Qiagen, Hilden, Germany) according to the manufacturer's instructions. The RNA samples were processed in triplicate at the Swegene Center for Integrative Biology Genomics DNA Microarray Resource Center (SCIBLU, Lund, Sweden) using MouseRef-8 Whole-Genome Expression Beadchips (Illumina, San Diego, CA, USA). Images and raw signal intensities were acquired using the Illumina BeadArray Reader scanner and Illumina BeadScan 3.5.31.17122 (Illumina, San Diego, CA, USA) image analysis software, respectively. Data preprocessing and quantile normalization were applied to the raw signal intensities using the web-based BioArray Software Environment system provided by SCIBLU. Further data processing was performed in Nexus Expression 2.0 (BioDiscovery, El Segundo, CA, USA) as previously described [20]. Differentially expressed transcripts (≥1.5-fold change) were identified with a Benjamini-Hochberg adjusted *p* value cutoff of <0.01, while enriched Gene Ontology (GO) terms associated with a gene set were identified using a *p* value cutoff of <0.05. The GO data obtained from the Nexus software were further analyzed and categorized using a web-based GO term search function [21,22]. Gene expression data discussed in this publication have been deposited at the NCBI's Gene Expression Omnibus [GEO:GSE32306].

The Human Protein Atlas and UniGene were used to establish normal expression of genes at the protein level in human thyroid and transcriptional level in mouse thyroid, respectively [23,24]. This analysis was only performed for differentially expressed genes common to all absorbed doses. In addition, the expression level of genes reported in the literature as potential biomarkers for retrospective dosimetry was investigated [13,14].

## Results and discussion

## Results

### General transcriptional changes

Distinct gene expression profiles were found in ^211^At-irradiated mouse thyroid tissue exposed to different absorbed doses (0.05, 0.5, 1.4, 11, or 32 Gy), compared to non-irradiated controls (Table 1 and Additional file 1). The highest number of differentially expressed transcripts (1,636 transcripts) was detected at 0.5 Gy, whereas the lowest absorbed dose (0.05 Gy) had a slightly lower impact on the transcriptome with 1,225 regulated transcripts. The number of regulated transcripts for the higher absorbed doses (1.4, 11, and 32 Gy) was 544, 575, and 425, respectively. Furthermore, down-regulation was predominantly observed at lower absorbed doses (0.05 and 0.5 Gy), and up-regulation, at higher absorbed doses (11 and 32 Gy). An intermediate phase was observed at 1.4 Gy, where an almost equal number of suppressed and induced transcripts were detected.

**Table 1:** Number of regulated transcripts

**Absorbed dose to the thyroid (Gy)**	**Number of differentially expressed transcripts**	**Number of up- and down-regulated transcripts**
0.05	1,225	↑ 338
		↓ 887
0.5	1,636	↑ 480
		↓ 1,156
1.4	544	↑ 284
		↓ 260
11	575	↑ 412
		↓ 163
32	425	↑ 293
		↓ 132

Total number of regulated (up- and down-regulated) transcripts in mouse thyroid glands after exposure to ^211^At. Up arrow, up-regulated transcript; down arrow, down-regulated transcript.

**Figure 1:**
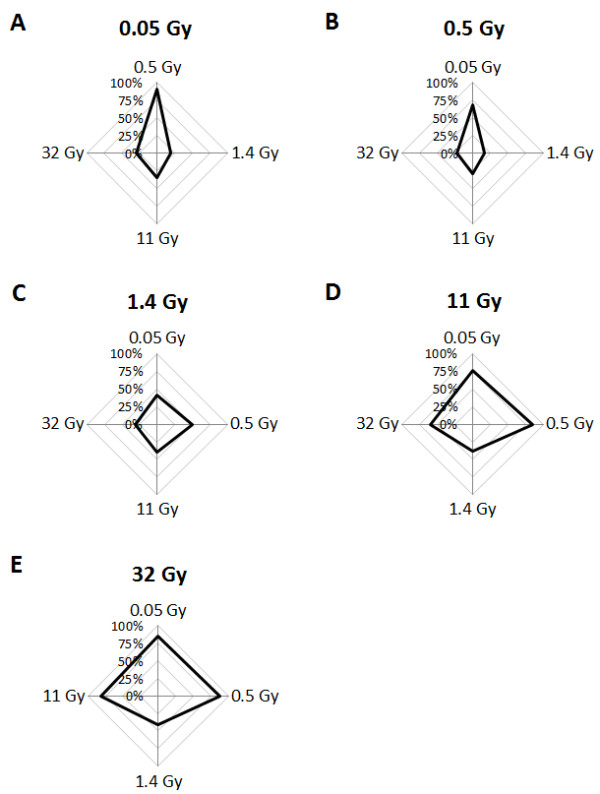
**Proportion of shared genes at the different absorbed dose levels.** The distribution of regulated transcripts is shown in a radar plot for the different absorbed dose levels: **(A)** 0.05 Gy, **(B)** 0.5 Gy, **(C)** 1.4 Gy, **(D)** 11 Gy, and **(E)** 32 Gy. For example, in (A), 91%, 18%, 36%, and 29% of the transcripts regulated at 0.05 Gy were also regulated at 0.5, 1.4, 11, and 32 Gy. For the exact distribution of regulated transcripts, see Additional file 2.

A high proportion of transcripts affected after exposure to 0.5 and 1.4 Gy were dose-specific (25% and 40%, respectively), whereas less than 8% of the transcripts detected at 0.05, 11, and 32 Gy were dose-specific (Figure 1 and Additional file 2). The high prevalence of down-regulation at the lowest absorbed doses (0.05 and 0.5 Gy) was common and unique for these treatment groups, among which 37% and 50% of the transcripts were down-regulated, respectively (Additional file 2).

### Effects of ^211^At irradiation on biological processes

Significantly expressed transcripts were associated to biological processes through gene ontology terms using the Nexus software (Table 2 and Additional file 3). The number of affected biological processes was highest at 1.4 Gy, with 27 affected processes. The affected biological processes were classified into seven broad GO terms: (1) immune response, (2) response to stimulus, (3) metabolism, (4) developmental process, (5) transport, (6) cellular process, and (7) system process (Table 2). Both 0.05 and 11 Gy induced an immunological response by regulating a general immune response (0.05 Gy), promoting thymic T cell selection (0.05 Gy), and by affecting B cell proliferation (11 Gy). In particular, absorbed doses of 0.5, 11, and 1.4 Gy had an impact on cellular response to stimulus, where 1.4 Gy affected biological processes connected to response to external stress. Exposure to 1.4 and 11 Gy also affected biological processes and signaling pathways involved in cell cycle regulation and proliferation (1.4 Gy: small GTPase signal transduction and transmembrane receptor protein tyrosine kinase signaling pathway; 11 Gy: G protein signaling and G protein receptor protein signaling pathway). An effect on metabolism was observed in all irradiated groups; however, 0.5 Gy affected both the highest number of unique and nonunique metabolic processes. Compared to 0.05, 0.5, and 32 Gy, absorbed doses of 1.4 and 11 Gy had a higher impact on developmental processes, e.g., differentiation and bone mineralization. Effects on transport of inorganic compounds were observed at all absorbed doses, while transport of organic compounds was only detected after exposure to 0.5 and 1.4 Gy.

**Table 2:** Affected biological processes (GO terms) categorized after function

**Biological process**	**Absorbed dose (Gy)**				
	**0.05**	**0.5**	**1.4**	**11**	**32**
Cellular process	4	1	1	2	2
Collagen fibril organization	✓				
Cytolysis	✓				
Epithelial cell differentiation	✓				
Mitochondrion organization and biogenesis	✓				
Negative regulation of endothelial cell proliferation		✓			
Cytoskeleton organization and biogenesis			✓	✓	✓
Cell migration				✓	
Negative regulation of microtubule depolymerization					✓
Immune response	2	0	0	1	0
Positive regulation of immune response	✓				
Positive thymic T cell selection	✓				
B cell proliferation				✓	
Metabolic process	7	13	7	5	4
Protein amino acid glycosylation	✓				
Regulation of metabolism	✓				
Lipid metabolism	✓	✓			
Metabolism	✓	✓			
Regulation of peptidyl-tyrosine phosphorylation	✓	✓			
Tricarboxylic acid cycle	✓	✓			
Ubiquinone biosynthesis	✓	✓			
Cellular protein metabolism		✓			
Cholesterol biosynthesis		✓			
Electron transport		✓			
Fatty acid metabolism		✓			
Glycolysis		✓			
Hormone metabolism		✓			
Protein folding		✓			
Acetyl-CoA biosynthesis from pyruvate		✓			
Amino acid biosynthesis			✓		
Retinoid metabolism			✓		
Signal peptide processing			✓		
tRNA processing			✓		
Peptidoglycan metabolism			✓		
Thyroid hormone generation			✓	✓	
One-carbon compound metabolism			✓		✓
Cholesterol metabolism				✓	
Steroid metabolism				✓	
Phosphocreatine metabolism				✓	✓
Lipopolysaccharide biosynthesis				✓	✓
Carbohydrate metabolism					✓
Transport	3	5	6	3	3
Oxygen transport	✓				
Secretion	✓	✓			
Potassium ion transport	✓	✓	✓	✓	✓
Protein transport		✓			
Transport		✓	✓		
Ion transport		✓	✓	✓	✓
Glucose transport			✓		
Iron ion transport			✓		
Vesicle-mediated transport			✓		
Diuresis				✓	
Sodium ion transport					✓
Response to stimulus	0	1	5	2	0
Response to unfolded protein		✓			
Chemotaxis			✓		
Response to cold			✓		
Response to hypoxia			✓		
Small GTPase signal transduction			✓		
Transmembrane receptor protein tyrosine kinase SP			✓		
G protein signaling				✓	
G protein receptor protein signaling pathway				✓	
System process	2	2	2	3	2
Muscle contraction	✓	✓		✓	✓
Regulation of muscle contraction	✓	✓	✓	✓	✓
Vasoconstriction			✓		
Digestion				✓	
Developmental process	2	1	4	5	2
Vasculogenesis	✓				
Fat cell differentiation	✓	✓			
Brain development			✓		
Cellular morphogenesis during differentiation			✓		
Regulation of bone mineralization			✓		
Cartilage condensation			✓	✓	
Bone mineralization				✓	
Brown fat cell differentiation				✓	
Male gonad development				✓	
Mechanoreceptor differentiation				✓	✓
Muscle development					✓

Transcripts with different gene expression levels are connected to biological processes (*p* < 0.05) through Gene Ontology terms. The main terms (cellular process, immune response, metabolic process, transport, response to stimulus, system process, and developmental process) have been compiled using the Gene Ontology search function [21,22]. Numbers presented after the main terms represent the number of affected biological processes for each absorbed-dose profile.

### Common differentially expressed transcripts among irradiated groups

**Figure 2:**
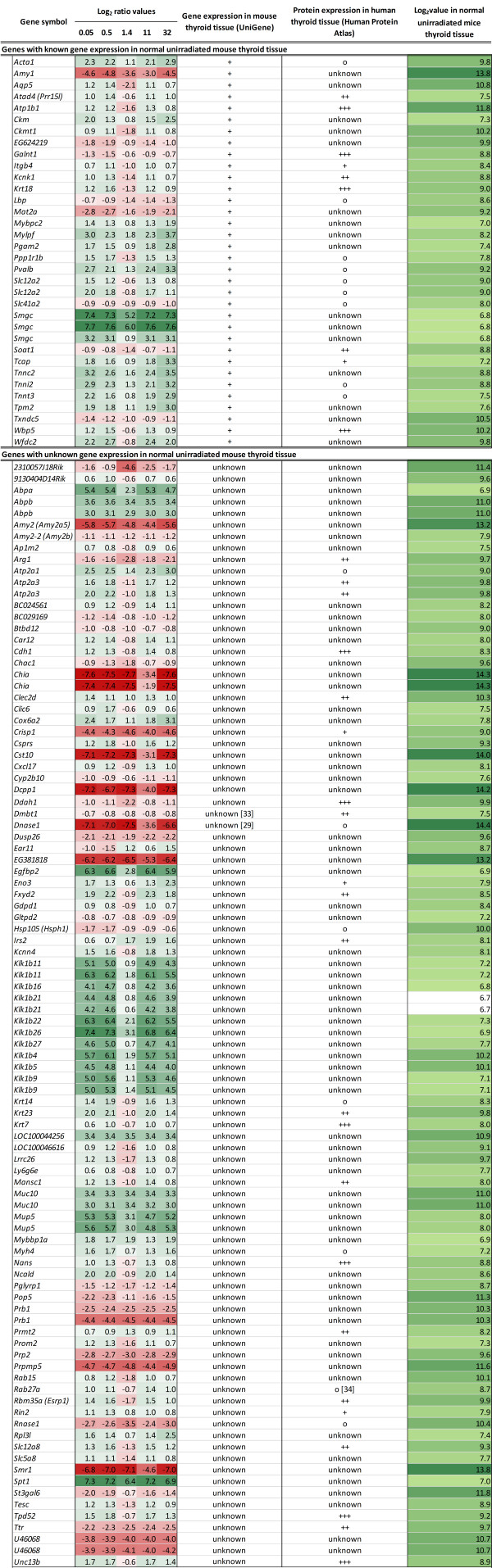
**Differentially expressed genes present at all absorbed doses.** Genes categorized by known gene expression in mouse thyroid according to UniGene and The Human Protein Atlas. Second column: genes are listed together with their log_2_ ratio value where the red color scale and the green color scale represent the magnitude of gene down-regulation and up-regulation, respectively. Genes annotated with ‘Unknown’ are genes with unknown gene and/or protein expression in normal thyroid tissue, and genes annotated with ‘o’ indicate that the gene is not expressed in normal thyroid tissue. For gene expression in mouse thyroid tissue, ‘+’ indicates gene expression, whereas for protein expression, ‘+’, ‘++’, or ‘+++’ indicates weak, moderate, or strong protein expression levels, respectively. The fifth column shows the log_2_ values of gene expression in non-irradiated mouse thyroids (the present study). Genes with a value equal to or greater than log_2_ 6.8 are considered as expressed in normal mouse thyroid.

In total, 130 differentially expressed transcripts (117 genes) were common for all absorbed doses (Figure 2). These play an important role in cellular metabolism, transport and communication, DNA, RNA and protein processing, immune response, apoptosis, cellular maintenance, and cell development. Of these, 40 transcripts (38 genes) were consistently up-regulated, and 46 transcripts (37 genes) were down-regulated in all groups. Interestingly, 43 transcripts (41 genes) associated with cellular transport, metabolism, and communication were down-regulated for 1.4 Gy, but up-regulated in all other groups. In addition, 12 transcripts representing nine genes associated with proteolysis (*Egfbp2*, *Klk1b11*, *Klk1b16*, *Klk1b21*, *Klk1b22*, *Klk1b27*, *Klk1b4*, *Klk1b5*, and *Klk1b9*) were present in highly elevated levels for 0.05, 0.5, 11, and 32 Gy (comparison log_2_ ratio, 3.6 to 6.6) but expressed to a lower extent for 1.4 Gy (comparison log_2_ ratio, 0.6 to 2.8).

Among the 130 shared transcripts, 12/34 transcripts normally expressed in mouse thyroids and 31/96 transcripts for which gene expression has not yet been determined in normal mouse thyroid were down-regulated after exposure to 1.4 Gy but up-regulated after exposure to 0.05, 0.5, 11, and 32 Gy (Figure 2). Furthermore, 18/31 transcripts with protein expression, 5/16 transcripts with no protein expression, and 20/83 transcripts having unknown protein expression in normal human thyroid tissue were up-regulated at the RNA level in mouse thyroids after exposure to 1.4 Gy, but down-regulated at the other four absorbed doses.

The *Dnase1* and *Rnase1* genes were strongly suppressed at the RNA level at all absorbed doses and have, according to UniGene and The Human Protein Atlas, unknown gene expression and no protein expression in normal mouse and human thyroid tissue, respectively. In addition, the *Ttr* (carrier of the thyroid hormone thyroxine) and *Crisp1* genes, normally expressed in human thyroid tissue, were down-regulated at all absorbed doses compared to non-irradiated controls.

To determine the number of normally expressed transcripts in mice thyroid, the expression levels in non-irradiated mice were investigated. The expression levels of genes with known mRNA expression in the thyroid ranged from log_2_ value 6.8 to 13.8 in non-irradiated controls (Figure 2). Among the shared genes with unknown expression at the mRNA level in mice thyroid tissue, all but one (*Klk1b21*) had a value higher than 6.8. Furthermore, 13,975 (10,578 genes) of the total number of 25,683 probes represented on the Illumina Beadchip had a log_2_ value higher than 6.8 (Additional file 4).

**Figure 3:**
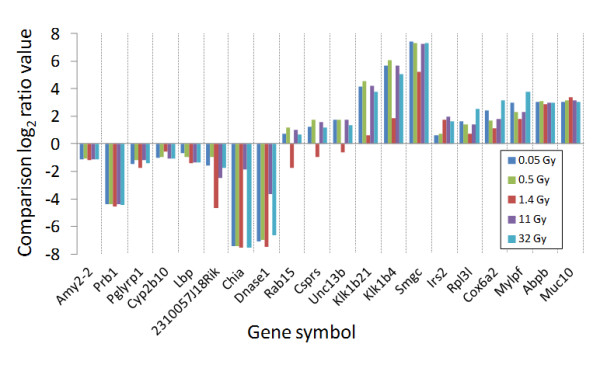
**Gene expression patterns for a selection of genes vs. absorbed dose.** A selection of genes chosen in order to illustrate the different gene expression patterns among the 130 differentially expressed genes at all absorbed doses studied. Values for gene expression are given as comparison log_2_ ratio values related to non-irradiated tissue.

The dose–response relationship for the expression level of the 130 shared transcripts exhibited different patterns (Figure 3). The expression levels of *Amy2-2*, *Prb1*, *Abpb*, and *Muc10* were clearly dose independent. The *Rab15*, *Csprs*, and *Unc13b* genes were suppressed for 1.4 Gy and up-regulated for 0.05, 0.5, 11, and 32 Gy, while *Klk1b21*, *Klk1b4*, and *Smgc* were more over-expressed for 0.05, 0.5, 11, and 32 Gy compared to 1.4 Gy. The *Chia* and *Dnase1* genes involved in apoptosis and immune response were highly suppressed at all absorbed doses but less suppressed at 11 Gy. The *Rpl3l*, *Cox6af*, and *Mylpf* genes had a u-shaped gene expression profile with high up-regulation for 0.05 and 32 Gy, less up-regulation for 0.5 and 11 Gy, and the least up-regulation for 1.4 Gy. The *Irs2* gene displayed two levels of gene expression: slight increase in gene expression for lower absorbed doses (0.05 and 0.5 Gy) and higher increase in gene expression for higher absorbed doses (1.4, 11, and 32 Gy).

### Changes in genes associated with thyroid function

Genes associated with ion transport were differentially expressed throughout the material. The Solute carrier family 5 member 8 (*Slc5a8*) gene, which is involved in controlling transmembrane iodine transport, was suppressed in thyroids irradiated with 1.4 Gy but present at elevated levels in all other groups. Several other genes belonging to the solute carrier family were also differentially expressed (Additional file 1).

The Ttr and Urah (synonym: 1190003j15rik) genes, both associated with thyroid hormone transport, were differentially expressed after irradiation at all absorbed doses, and at all absorbed doses except 32 Gy, respectively. While Ttr was consistently down-regulated, Urah was down-regulated at 1.4 Gy and up-regulated at 0.05, 0.5, and 11 Gy.

### Comparison with previously known radiation-associated genes

Few radiation-specific genes from the literature were differentially expressed in mouse thyroids 24 h following injection of ^211^At (Table 3) [13,14]. The *Amy1* and *Amy2* genes, which have unknown functions in the thyroid, were both substantially suppressed at all absorbed doses compared to non-irradiated controls. The *Ccnd1* and *Gjb2* genes were expressed in the opposite direction at 1.4 Gy when compared with 0.5 Gy and 0.05, 0.5 and 11 Gy, respectively. Several genes associated with the tumor necrosis factor superfamily (*Tnfaip8*, *Tnfrsf19*, and *Tnfrsf21*) were slightly up-regulated at various absorbed doses. The *Trp53inp1* gene was up-regulated at 0.05, 1.4, 11, and 32 Gy, while *Trp53inp2* was down-regulated at 0.5 Gy. Both genes are associated with response to double-strand DNA breaks and apoptosis induction.

**Table 3:** Radiation-associated genes

**Gene symbol**	**Log**_**2**_**ratio values**				
	**0.05 Gy**	**0.5 Gy**	**1.4 Gy**	**11 Gy**	**32 Gy**
*Amy1 (Mycbp)*	−4.6	−4.8	−3.6	−3.0	−4.5
*Amy2*	−5.8	−5.7	−4.8	−4.4	−5.6
*Apaf1*					
*Bag-1*					
*Batf3*					
*Bax*					
*Ccnb1*					
*Ccnd1*		0.6	−0.9		
*Ccne1*					
*Ccng1*					
*Cdc25*					
*Cdkn1a*		−0.6			
*Dd2*					
*Ddb2*					
*Ephx2*		−0.8			
*Fen1*					
*Fhl2*					
*Flt3*					
*Fos*			1.1		
*Gadd45a*			0.8		
*Gadd45g*	0.6		1.1		
*Gja1*		−1.2			
*Gjb2*	0.7	0.9	−0.9	0.8	
*Hiap*					
*Hrad23b*					
*Iap3*					
*Icsbp1*					
*Jun*					
*Xrcc6 (Ku70)*	−0.7	−0.9			
*Mak10*					
*Makp8pi*					
*Mapk1*					
*Mapk3*					
*Mdm2*					
*Noxa*					
*Ogg1*					
*Pcna*					
*Plcg2*		0.7		0.7	
*Rad51l1*					
*Rhoa*					
*Tgfb3*		1.0			
*Tgfbr2*	−0.7	−0.9			
*Tnfaip8*			0.7		
*Tnfrsf10b*					
*Tnfrsf19*	0.7	0.6		0.7	
*Tnfrsf21*	0.7	1.0		0.9	0.6
*Tp53*					
*Trp53inp1*	0.7		0.8	0.9	0.7
*Trp53inp2*		−0.6			
*Ttf*					
*Wee*					
*Vegfb*		−0.8			
*Xab2*					
*X-iap*					
*Xpc*					
*Xrcc1*					

Genes associated with radiation-induced biological response according to Snyder and Morgan [13] and Chaudhry [14]. Values for gene expression are given as comparison log_2_ ratio values. Note that the Illumina MouseRef-8 Beadchip is a whole-genome expression assay; however, some gaps in the genome do exist, and several genes are represented under other aliases.

## Discussion

The present study represents a multifaceted and extensive approach to investigate the transcriptional changes in normal mouse thyroid tissue after internal exposure to ^211^At. A broad range of absorbed doses were used, from 0.05 to 32 Gy delivered over 24 h, in order to make the study valid for both environmental and clinical practices.

The results show that the mouse thyroid gland responds to radiation in an absorbed-dose-dependent manner but with few common differentially expressed genes and affected biological functions at all absorbed doses studied. The most surprising results were the wide biological response involving a large set of genes differentially expressed between different absorbed doses and that few of the genes currently known to be associated with cellular radiation damage were differentially expressed, which implies an organ-specific response to ionizing radiation.

Exposure to ^211^At resulted in a strong transcriptional response in mouse thyroid tissue, with the most substantial impact on the transcriptome at the two lowest absorbed doses studied (0.05 and 0.5 Gy). To the best of our knowledge, no studies to date (*in vivo*, *ex vivo*, or *in vitro*) have compared the number and the type of differentially expressed transcripts over such a wide absorbed-dose range. This makes it difficult to compare and validate the distribution and specificity of the differentially expressed transcripts identified in this study. Consequently, the present study may function as a valuable foundation for future studies similar to this one.

Few differentially expressed genes were common for the different absorbed doses. Similarities were mainly observed when comparing 0.05 with 0.5 Gy. From the distribution of differentially expressed transcripts, it is evident that, at low absorbed doses where a similar transcriptional response was anticipated, the response shifts (the number and direction of regulated genes) as the absorbed dose increases from 0.5 to 1.4 Gy. In addition, although 1.4 Gy had the highest proportion of non-shared transcripts compared with the other absorbed doses, the pooled response at 0.05 and 0.5 Gy was even more unique. Together, these findings indicate that there is an underlying fundamental difference in the cellular response to radiation after low-dose irradiation compared to high doses with an intermediate phase at 1.4 Gy.

Gene ontology data provide comprehensive descriptions of defined gene product properties, which describe how genes function in a normal cellular context. The Nexus software is quite specific and does not classify regulated biological functions into main categories. However, a broader categorization was performed using a web-based GO term search function [21,22]. In the present investigation, the regulated biological functions were classified under seven different main biological processes (cellular process, immune response, metabolic process, transport, response to stimulus, system process, and developmental process). Biological processes related to cell signaling and response to stimuli with an impact on cell cycle regulation and proliferation were activated through the signal transduction pathway and only detected at 1.4 and 11 Gy. Biological processes related to transport were affected at all absorbed doses with a peak at 1.4 Gy. Together with regulation of signal transduction at 1.4 Gy, this indicates that there is a high activity of activated cellular processes in the thyroid. We suggest that this is a radiation-induced response to damages in the DNA and/or other sub-cellular components from absorbed doses high enough to produce a severe but still reparable damage. At absorbed doses above 11 Gy, lesions are probably too large to be repaired, resulting in reduced cellular activity to primarily maintain survival since some transport and signal transduction systems are still working to some extent. Immune response was observed in thyroid tissue after exposure to 0.05 and 11 Gy. However, no biological processes related to inflammatory response were detected at any absorbed doses. These results are in agreement with a previous study on gene expression levels in mouse brain and kidney tissue where an inflammatory response was not triggered 8 and 24 h after irradiation [15]. Inflammation is a primary acute response to radiation damage with regulation of chemokines and cytokines minutes to hours subsequent irradiation; after which, a more proliferative phase begins with cell migration and macrophage and monocyte activity [2]. Since the present study has been performed with internal ongoing irradiation with maximum uptake of ^211^At at 16 h after injection, killing the animals 24 h following irradiation might be too late a time point to observe regulation of chemokines and cytokines and too early for late inflammatory effects. To further study the inflammatory response at a transcriptional level, studies with different temporal end points are needed. In addition, metabolic, system, and developmental processes are regulated at all absorbed doses. We suggest that these are related to basal cellular maintenance functions.

Radiation damage will eventually be healed through tissue remodeling, and an excessive deposition of collagen is a known characteristic of radiation fibrosis. Collagen synthesis usually occurs months to years after radiation damage; hence, effects related to collagen were not likely to be apparent in the present study [2]. However, an effect on collagen fibril organization was observed after exposure to 0.05 Gy, indicating fibrogenesis activity. In addition, up-regulation of the pro-inflammatory tumor necrosis factor alpha (*Tnfa)* is an early sign of radiation-induced fibrogenesis [25]. Activation of *Tnfaip8* was observed at 1.4 Gy alone, but genes associated with tumor necrosis factor receptors were up-regulated at all absorbed doses except for 1.4 Gy.

Statistically, a mean absorbed dose of 1.2 Gy results in one alpha particle track per thyroid cell, which is close to the 1.4 Gy used in this study. Consequently, 0.05 and 0.5 Gy will result in hitting only approximately 4% and 40% of the cells in the thyroid gland (A Josefsson and E Forssell-Aronsson, unpublished work). The increase in absorbed dose from 0.05 and 0.5 Gy to 1.4 Gy should, therefore, not mainly result in an increase of energy deposited in each cell but rather in an increased number of cells hit. If this is true, the biological effects reflected by the gene expression seen at 0.05 and 0.5 Gy would be diluted (the same transcripts affected, but with lower magnitude) as the majority of the cells participating in the analysis are not actually irradiated (but still included in the analysis). In addition, at 0.05 and 0.5 Gy, the complete thyroidal response involves a much higher number of genes compared to when a higher proportion of cells are irradiated. These results could be explained by a bystander effect, with regulation (activation and/or repression) of a substantial number of genes at lower absorbed doses with origin in non-irradiated cells. Gene expression studies of the bystander effect *in vitro* have shown that non-irradiated cells exposed medium from irradiated cells primarily induced over-expression of a large number of genes [26]. This is not consistent with our data as the majority of genes detected only at 0.05 and 0.5 Gy were primarily down-regulated. Another study used a low fluency of alpha particles and demonstrated that, when only 2% of the cell population was irradiated, the *CDKN1A*, *CDC2*, *CCNB1*, and *RAD51* genes were expressed in a greater proportion of cells than anticipated, an effect that decreased in the presence of a gap junction inhibitor [27]. None of these genes were affected in the present study with the exception of *Cdkn1a*, which was down-regulated after exposure to 0.5 Gy. The lack of response in these genes may be related to the timing of the experiment.

As a result of working with an animal model, the injected ^211^At will circulate throughout the body and, thus, irradiate organs other than the thyroid. As a consequence of organ interaction (through, e.g., signaling and hormones), some of the biological response seen in the present study may originate from tissues other than the thyroid. A previous study using ^131^I demonstrated that very low absorbed doses (in milligray level) had a distinct impact on gene expression in the liver, spleen, kidneys, and lung 24 h after injection [28]. Assuming that this also occurs after ^211^At exposure, signaling substances emitted from other organs in the mouse might have an impact on gene expression in the thyroid.

To fully understand the cellular response to ionizing radiation, it is necessary to have a comprehensive picture of the normal expression of genes and different signaling pathways involved in each specific tissue. Therefore, we identified normally expressed genes in normal mouse thyroid tissue by comparing mRNA expression levels for transcripts with previously known and unknown expression patterns in mouse thyroid [29]. However, compared to expression in normal thyroid tissue, genes down-regulated in irradiated thyroid tissue must have some expression at the transcriptional level in normal untreated thyroid tissue. The shared genes with known expression at the mRNA level in normal mouse thyroid tissue had a log_2_ value between 6.8 and 13.8 in non-irradiated mouse thyroid tissue. We used the lower log_2_ value as a cutoff where all genes with a log_2_ value over 6.8 were considered as normally expressed. All but one of the genes (*Klk1b21*) that were differentially expressed at all absorbed doses were expressed in normal mouse thyroid. By using this method, we identified 10,578 transcripts in the present study with elevated mRNA levels.

Thyroid stunning is a debated phenomenon where a low amount of radioiodine administered for treatment planning results in a lower thyroid uptake of administered radioiodine at the subsequent treatment [30]. The gene *SLC5A5* encodes for the sodium-iodide symporter, which is responsible for transporting iodine and, to some extent, astatine into the thyroid follicles [12]. Transport of iodine after ^211^At exposure has been measured in an *in vitro* system using thyroid cells where an increase in iodine transport was reported immediately after irradiation ended. At 12 h, the iodine transport returned to normal only to further decrease over the following 5 days. Down-regulation at the mRNA level of NIS was 61% at 5 days after irradiation compared with non-irradiated controls [31]. In humans, the *SLC5A8* gene encodes for the sodium/monocarboxylate transporter, which is 70% homologous to NIS [32]. In the present study, the *Slc5a8* gene was down-regulated at 1.4 Gy, but up-regulated at all other absorbed doses. Several other genes that belong to the solute carrier group were also differentially expressed, but their impact on iodide transport is still unknown. Additionally, the *Ttr* gene (down-regulated for 1.4 and 11 Gy) encodes for the transthyretin protein, which takes part in transporting thyroxine (T4). Little is known about the expression of transthyretin in human thyroid tissue, and it is mainly produced in the brain and liver. Taken together, the thyroid function is affected at some level; however, the impact it may have at a higher translational level is unclear. In addition, we suggest that future studies should include measurements of the thyroid stimulating hormone blood levels as well as other suitable biomarkers of thyroid function.

The 130 transcripts differentially expressed and shared among all irradiated groups may be attributed to (1) genes with normal expression in thyroid tissue, (2) genes not normally expressed but still specific for thyroid tissue, and (3) genes that are specific for ^211^At irradiation. However, as majority of these transcripts have unknown protein expression in human thyroid tissue (83/130) and unknown gene expression in mouse thyroid tissue (96/130) (according to The Human Protein Atlas and UniGene, respectively), the possibility to determine this distribution is limited. Furthermore, not corresponding with UniGene and The Human Protein Atlas, *Dmbt1* (synonym: *Crp*) and *Rab27a* are expressed at the mRNA level in mouse thyroid and at the protein level in human thyroid, respectively [33,34]. In addition, *Dnase1* has detectable mRNA levels in mouse thyroid [29]. Also, several highly affected genes detected in the present study have unknown biological functions, such as *Spt1* (up-regulated with fold change: 152, 152, 83, 143, and 123 at 0.05, 0.5, 1.4, 11, and 32 Gy, respectively) and *Smr1* (down-regulated with fold change: 115, 132, 138, 24, and 128 at 0.05, 0.5, 1.4, 11, and 32 Gy, respectively). These genes may play an important role in the biological response to irradiation, and further studies at the protein level will possibly clarify their involvements.

In a clinical phase-I study, patients were infused with ^211^At-labeled monoclonal antibody fragments (absorbed doses were 0.2 to 0.82 Gy and 0.02 to 0.18 Gy to the unblocked and blocked thyroid, respectively) without detecting any toxicological effects after 23 months [10]. These absorbed doses are close to the 0.05- and 0.5-Gy doses used in the present study. Many cellular processes and a high number of transcripts were regulated after exposure at these low absorbed doses. We deem it important to perform molecular studies on radiation-induced changes in the thyroid at different temporal end points in order to gain a more in-depth understanding of possible damaging effects.

Gene expression profiling has been proposed as a potential biomarker for retrospective biodosimetry, and studies have been performed primarily *in vitro* and *ex vivo* using X-rays or gamma radiation [13,14]. The present study does not validate the genes suggested as candidate biomarkers in these reviews since a minor proportion were differentially expressed in our data (Table 3). However, there are many differences between internal alpha particle irradiation from radionuclides and external irradiation, where a linear accelerator or gamma-emitting radionuclides are used. Additionally, *in vitro* and *ex vivo* studies do not provide a comprehensive explanation of the causal effects of radiation in tissues as these methods lack the complexity of a complete organism with interacting tissues. Thyroid tissue consists of many different cell types, both tissue-specific (thyrocytes and C cells) and general cells (e.g., endothelial, muscular, and fat cells). It is not likely that a single gene will be able to function as a retrospective biomarker for biodosimetry; however, computational informatics have been used to construct a 74 multi-genetic signature that can be used to distinguish between four different absorbed doses (0.5, 2, 5, and 8 Gy using Cs-137 as a gamma source), an approach which this present study supports [35].

## Conclusions

In conclusion, these results demonstrate the complexity of radiation-induced biological effects. Thus, care should be taken when interpolating effects between high and low absorbed doses, independent of gene expression level. We conclude that the thyroid was significantly affected at absorbed-dose levels similar to those obtained in radionuclide therapy for tumors. Due to the increased heterogeneous irradiation at low absorbed doses, we suggest that this response partly originates from non-irradiated cells in the tissue, i.e., bystander cells.

## Competing interests

The authors declare that they have no competing interests.

## Authors' contributions

NR and EFA designed the animal study, while NR and TZP carried out the animal trial. TZP carried out the extraction of RNA and preprocessing of the data. NR performed the first analysis of the data along with KH. All authors contributed to the scientific and intellectual discussion and interpretation of the data. NR drafted the manuscript; however, all authors participated with substantial input and revision of the manuscript. All authors read and approved the final manuscript.

## Authors' information

NR has an MSc degree in physics, ES has an MSc degree in medical physics, and TZP has a BSc degree in biology. They are PhD students at the Sahlgrenska Academy, which is the medical faculty of the Gothenburg University. KH has a PhD degree in genetics and is an associate professor in genetics. EFA has a PhD degree in medical physics and is a professor in medical physics.

## Supplementary Material

Additional file 1 **Comparisons between all irradiated and non-irradiated groups.** Raw data is given from the Nexus program, which compares gene expression levels between two selected groups. Here, every possible comparison is shown. Click here for file

Additional file 2 **The distribution of differentially expressed transcripts.** The differentially expressed transcripts are divided into ‘sets’, which describe similarities and differences among the absorbed doses. Here, these sets are shown with the number and percentage of the total number of regulated transcript for each absorbed dose. Red color represents down-regulation, green color represents up-regulation, and the blue color scale to the right represents the magnitude of percent transcripts distributed in that specific set for each absorbed dose. For example, six transcripts (equal to 0.5% of the total number of 1,225 transcripts regulated after exposure to 0.05 Gy) are up-regulated and unique for the 0.05-Gy gene expression profile. Click here for file

Additional file 3 **Affected biological processes with associated genes.** Regulated genes are connected to biological processes through Gene Ontology terms. Here, all affected biological processes are shown together with their regulating genes. Gene expression is either induced or repressed while direction of regulation of the biological processes is not accessible. Click here for file

Additional file 4 **Log**_**2**_**values for all 25,683 probes used.** Genes with log_2_ value over 6.8 is considered to be expressed in normal unirradiated mouse thyroid and marked green. Genes marked red are not expressed in normal mouse thyroid. Data is sorted from A to Z. Click here for file
